# Evaluation of glutathione S-transferase P1 (GSTP1) Ile105Val polymorphism and susceptibility to type 2 diabetes mellitus, a meta-analysis

**DOI:** 10.17179/excli2017-828

**Published:** 2017-11-06

**Authors:** Mostafa Saadat

**Affiliations:** 1 Department of Biology, College of Sciences, Shiraz University, Shiraz 71467-13565, Iran

**Keywords:** susceptibility, heterogeneity, risk, Diabetes mellitus, genetic polymorphism

## Abstract

It is well established that type 2 diabetes mellitus (T2DM) is associated with oxidative stress and glutathione S-transferases (GSTs) protect cells against oxidative stress. The missense substitution Ile105Val (rs1695) of the glutathione S-transferase P1 (GSTP1, OMIM: 134660) results from an A/G base substitution at nucleotide 313. Many studies have evaluated the correlation between the rs1695 polymorphism and T2DM, but the results remain inconclusive. The aim of the present meta-analysis was to investigate the association between GSTP1 Ile105Val polymorphism and the susceptibility risk of T2DM. Eligible studies (published before August 2017) were identified in several databases. The heterogeneity between studies was evaluated with the chi-square based Q test and the I2 test. The strengths of the association were assessed by pooled odds ratios (ORs) and the corresponding 95 % confidence interval (95 % CI) using either a fixed or random-effects models. Eighteen studies documenting a total of 2595 T2DM cases and 2888 controls were included in this meta-analysis. In the overall analysis there was no significant association between the rs1695 polymorphism and the risk of T2DM. The subgroup analyses stratified by ethnicity, publication year and sample size did not reveal significant association between the study polymorphism and the risk of T2DM and any sources contributing to the substantial heterogeneity between studies. The present meta-analysis suggested that there was significant heterogeneity between studies. Considering some limi tations of our meta-analysis, further large-scale studies should be done to reach a more comprehensive understanding.

## Introduction

Oxidative stress defines as imbalance between reactive oxygen species and anti-oxidant defense systems and it is associated with pathogenesis of diseases. In human, genes involved in antioxidant defenses are highly polymorphic and show association with several multifactorial traits (Saadat, 2006[25], 2013[26]; Saadat and Saadat, 2015[27]; Kang, 2015[15]; Jamhiri et al., 2017[13]; Zendehboodi, 2017[37]). 

It is well established that oxidative stress plays an important role in the development of type 2 diabetes mellitus (T2DM) (Asmat et al., 2016[5]). The prevalence of T2DM is rising rapidly in both developed and developing countries (Ginter and Simko, 2012[9]; Sun et al., 2014[30]). Many studies point to that genetic predisposition plays an important role in the development of T2DM (Almgren et al., 2011[3]; Sun et al., 2014[30]; Stančáková and Laakso, 2016[28]). 

One of the most important cellular detoxification systems is glutathione S-transferase (GST, EC 2.5.1.18) enzymes. The GSTs are involved in catalyzing the conjugation reactions of reactive intermediates of electrophilic compounds with cytosolic glutathione. The glutathione S-transferase P1 (GSTP1, OMIM: 134660) encodes the π-class of GSTs, which accounts for about 90 % of the enzymatic activity of the GST family; and expressed in many normal tissues (Townsend and Tew, 2003[32]). 

Human *GSTP1* is polymorphic and its variant alleles occurr at a high frequency. The missense substitution Ile105Val (rs1695) of the *GSTP1* results from an A/G base substitution at nucleotide 313. The Val105 form of the GSTP1 enzyme may be 2-3 times less stable than the Ile105 form (Johansson et al., 1998[14]) and may be associated with a higher level of DNA adducts (Ryberg et al., 1997[24]). Enzymes with the valine at amino acid 105 have a seven-fold higher catalytic efficiency for the diol epoxides of polycyclic aromatic hydrocarbons than the isoenzymes with the isoleucine at this position. In contrast, the Val105 enzyme is three-fold less effective with 1-chloro-2,4-dinitrobenzene as a substrate (Zimniak et al., 1994[38]; Hu et al., 1997[12]; Sundberg et al., 1998[31]).

The association between the Ile105Val *GSTP1* polymorphism and the risk of T2DM has been investigated, but these studies yielded controversial results (Yalin et al., 2007[35]; Oniki et al., 2008[20]; Bid et al., 2010[6]; Tsai et al., 2011[33]; Ramprasath et al., 2011[21]; Amer et al., 2012[4]; Moasser et al., 2012[19]; Gönül et al., 2012[10]; Grubisa et al., 2013[11]; Mastana et al., 2013[17]; Vats et al., 2013[34]; Rao et al., 2014[22]; Abbasi et al., 2014[1]; Zaki et al., 2015[36]; Stoian et al., 2015[29]; Mergani et al., 2016[18]; Rasheed et al., 2016[23]; Ahmed and Al-Bachary, 2017[2]). Some studies suggested that the *GSTP1* polymorphism is associated with susceptibility to T2DM (Bid et al., 2010[6]; Amer et al., 2012[4]; Mastana et al., 2013[17]; Vats et al., 2013[34]; Rao et al., 2014[22]; Zaki et al., 2015[36]; Stoian et al., 2015[29]; Mergani et al., 2016[18]), other reports, however, do not support the finding (Yalin et al., 2007[35]; Oniki et al., 2008[20]; Tsai et al., 2011[33]; Moasser et al., 2012[19]; Gönül et al., 2012[10]; Grubisa et al., 2013[11]; Abbasi et al., 2014[1]; Rasheed et al., 2016[23]). Whether *GSTP1* polymorphism modifies the risk of T2DM remains uncertain, therefore the present meta-analysis was carried out.

## Methods

### Search strategy

Literature databases, including PubMed, Scopus, DOAJ, Index Copernicus, JSTOR, JournalTOCs, AJOL (African Journal Online), Asia Journal Online, Index Scholar, Academic Journals Databases, Google Scholar, Research Bib, High-Wire, J-STAGE, Serbian citation index, KoreaMed, IndMed, PakMediNet and SID (Scientific Information Database) were searched for relevant studies (the last search was updated in August 2017). 

The following search terms were used: ('type 2 diabetes mellitus', 'glutathione S-transferase P1' '*GSTP1*', 'rs1695', polymorphism, Ile105Val. The search was limited to studies published in English or Persian. In addition the bibliographies of the retrieved studies were screened to identify relevant publications.

### Inclusion and exclusion criteria

The eligible studies had to meet the following criteria: (1) a case-control study to evaluate the association between rs1695 polymorphism and risk of T2DM; (2) raw data including sample size, allele or genotype distribution were given. Accordingly, the exclusion criteria were as follows: reviews, meta-analysis, editorial articles, abstracts, comments.

### Data extraction

The following information was extracted from each: Name of the first author, year of publication, country of origin, ethnicities of the individuals involved, number of cases and controls, genotyping method, and number of genotypes for the study polymorphism in cases and controls. 

### Statistical analysis

The crude odds ratios (ORs) and 95 % confidence intervals (95 % CIs) of *GSTP1* Ile105Val polymorphism and risk of T2DM were estimated for each study. Hardy-Weinberg equilibrium in controls was tested by a chi-square test. 

The heterogeneity between studies was evaluated with the chi-square based Q test and the I^2 ^test. An I^2^ value of less than 25 % indicates low heterogeneity, 25 % to 50 % indicates moderate heterogeneity, and greater than 50 % indicates high heterogeneity. I^2^> 50 % or P < 0.10 for Q statistics was considered as significant heterogeneity. 

If no significant heterogeneity was found between the studies, the pooled OR was calculated by using the fixed effects model (the Mantel-Haenszel method) (Mantel and Haenszel, 1959[16]). Otherwise, the random effects model (the DerSimonian and Laird method) was applied (DerSimonian and Laird, 1986[7]). Three genetic models were performed in the meta-analysis: codominant model (Ile/Val *vs* Ile/Ile and Val/Val *vs* Ile/Ile), dominant model (Ile/Val + Val/Val *vs* Ile/Ile) and allele model (Val *vs* Ile). We also performed subgroup analysis according to ethnicity (Caucasian), sample size (≤300/>300 subjects), and publication year (prior to or during 2012/after 2012). 

Moreover, sensitivity analysis was performed to evaluate the stability of the results following sequential removal of each study. To determine the evidence of publication bias, the funnel plot and Egger's test were both used. An asymmetric plot suggested possible publication bias. For the interpretation of Egger's test, statistical significance was defined as P<0.05 (Egger et al., 1997[8]). 

## Results

In total, 18 independent studies including 5483 subjects (2595 T2DM cases and 2888 controls) were included in the study. The studies were published between 2007 and 2017. The characteristics of each study were summarized in Table 1(Tab. 1) (References in Table 1: Yalin et al., 2007[35]; Oniki et al., 2008[20]; Bid et al., 2010[6]; Tsai et al., 2011[33]; Ramprasath et al., 2011[21]; Amer et al., 2011[4]; Moasser et al., 2012[19]; Gönül et al., 2012[10]; Grubisa et al., 2013[11]; Mastana et al., 2013[17]; Vats et al., 2013[34]; Rao et al., 2014[22]; Abbasi et al., 2014[1]; Zaki et al., 2015[36]; Stoian et al., 2015[29]; Mergani et al., 2016[18]; Rasheed et al., 2016[23]; Ahmed and Al-Bachary, 2017[2]). The studies included in the present meta-analysis were conducted on different ethnic populations: 14 studies included a Caucasian population, 2 studies included an Asian population, and 2 studies included an African population. In all studies the study polymorphism was evaluated by RFLP-PCR method.

**Figure 1:**
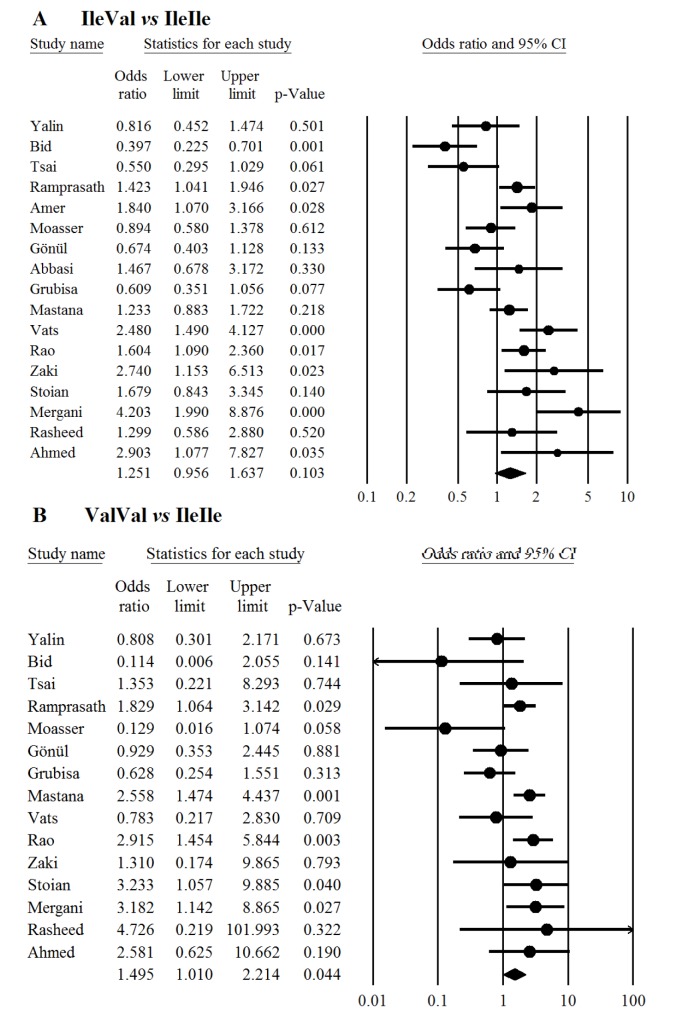
Meta-analysis of the *GSTP1* Ile105Val polymorphism with type 2 diabetes mellitus under codominant genetic model; Panels A and B represent Ile/Val *vs* Ile/Ile and Val/Val *vs* Ile/Ile respectively. Each box represents the odds ratio (OR) point estimate and its 95 % confidence interval (CI). The diamond represents the overall summary estimate, with confidence interval represented by its width. Pooled ORs and 95 % CI are estimated by Dersimonian and Laird random-effects model.

**Figure 2:**
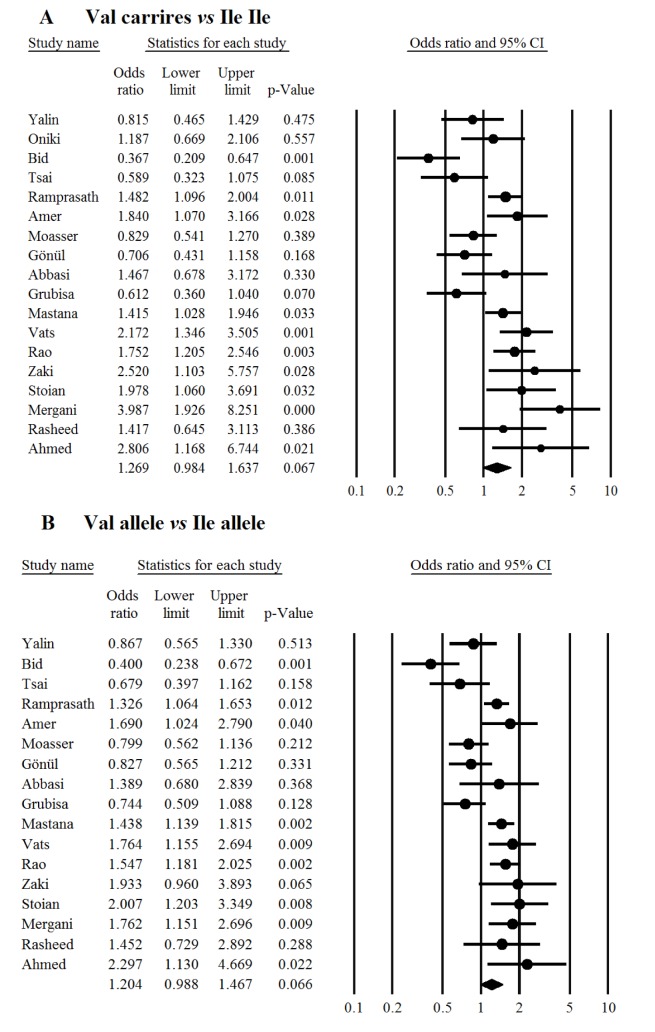
Meta-analysis of the *GSTP1* Ile105Val polymorphism with type 2 diabetes mellitus under dominant (A) and allele genetic models (B). Each box represents the odds ratio (OR) point estimate and its 95 % confidence interval (CI). The diamond represents the overall summary estimate, with confidence interval represented by its width. Pooled ORs and 95 % CI are estimated by Dersimonian and Laird random-effects model.

In overall there was no significant association between the study polymorphism and the risk of T2DM under codominant (Figure 1(Fig. 1); References in Figure 1: Yalin et al., 2007[35]; Oniki et al., 2008[20]; Bid et al., 2010[6]; Tsai et al., 2011[33]; Ramprasath et al., 2011[21]; Amer et al., 2011[4]; Moasser et al., 2012[19]; Gönül et al., 2012[10]; Grubisa et al., 2013[11]; Mastana et al., 2013[17]; Vats et al., 2013[34]; Rao et al., 2014[22]; Abbasi et al., 2014[1]; Zaki et al., 2015[36]; Stoian et al., 2015[29]; Mergani et al., 2016[18]; Rasheed et al., 2016[23]; Ahmed and Al-Bachary, 2017[2]), dominant (Figure 2A(Fig. 2); References in Figure 2: Yalin et al., 2007[35]; Oniki et al., 2008[20]; Bid et al., 2010[6]; Tsai et al., 2011[33]; Ramprasath et al., 2011[21]; Amer et al., 2011[4]; Moasser et al., 2012[19]; Gönül et al., 2012[10]; Grubisa et al., 2013[11]; Mastana et al., 2013[17]; Vats et al., 2013[34]; Rao et al., 2014[22]; Abbasi et al., 2014[1]; Zaki et al., 2015[36]; Stoian et al., 2015[29]; Mergani et al., 2016[18]; Rasheed et al., 2016[23]; Ahmed and Al-Bachary, 2017[2]) and allele genetic models (Figure 2B(Fig. 2)). It should be noted that there was significant heterogeneity in the examined genetic models for the rs1695 polymorphism (Table 2(Tab. 2); References in Table 2: Oniki et al., 2008[20]; Vats et al., 2013[34]; Rao et al., 2014[22]; Ahmed and Al-Bachary, 2017[2]). The genotyping frequencies in the control groups showed significant differences with the expected frequencies in three studies (Vats et al., 2013[34]; Rao et al., 2014[22]; Ahmed and Al-Bachary, 2017[2]). After excluding these studies from meta-analysis, no significant association between the Ile105Val polymorphism and the T2DM risk under examined models was observed (Table 2(Tab. 2)). Excluding 3 studies did not alter heterogeneity between studies. 

The source of heterogeneity was assessed by ethnicity, publication year, and sample size. The subgroup analyses did not reveal any sources contributing to the substantial heterogeneity (Table 2(Tab. 2)). In stratified subgroups based on ethnicity, publication year and sample size, no statistically significant association was observed between the *GSTP1* Ile105Val polymorphism and the risk of T2DM in any of the genetic models (Table 2(Tab. 2)).

Sensitivity analyses were performed by sequential omission of individual studies for all subjects and subgroups. The corresponding pooled ORs were not altered in all subjects and subgroups of *GSTP1* genotypes (data not shown). The results of sensitivity analyses indicated the stability of the results of this meta-analysis. Funnel plots were created and Egger's test was performed to assess the publication bias of the included studies. The funnel plots did not show obvious asymmetry in the overall population (data not shown). 

## Discussion

It has been well established that oxidative stress is involved in pathogenesis of T2DM (Sun et al., 2014[30]). Considering that GSTP1 belongs to GST superfamily and is involved in cellular detoxification and the fact that Ile105Val polymorphism may alter the GSTP1 enzyme activity (Zimniak et al., 1994[38]; Johansson et al., 1998[14]; Ryberg et al., 1997[24]; Hu et al., 1997[12]; Sundberg et al., 1998[31]), it seems that the rs1695 polymorphism may be associated with susceptibility to T2DM. During 2007 to 2017, eighteen studies investigate the association between this polymorphism and the risk of T2DM (Yalin et al., 2007[35]; Oniki et al., 2008[20]; Bid et al., 2010[6]; Tsai et al., 2011[33]; Ramprasath et al., 2011[21]; Amer et al., 2012[4]; Moasser et al., 2012[19]; Gönül et al., 2012[10]; Grubisa et al., 2013[11]; Mastana et al., 2013[17]; Vats et al., 2013[34]; Rao et al., 2014[22]; Abbasi et al., 2014[1]; Zaki et al., 2015[36]; Stoian et al., 2015[29]; Mergani et al., 2016[18]; Rasheed et al., 2016[23]; Ahmed and Al-Bachary, 2017[2]). However, the results of the studies are not consistent. Therefore we carried out a meta-analysis. In the present meta-analysis we found high level of heterogeneity between studies and no association between the examined genetic models and the risk of T2DM (Table 2(Tab. 2)).

Some limitations of the present meta-analysis should be acknowledged. The non-conformity of the geographical distribution of the association studies used in the meta-analysis is the most important limitation of the present study. There was no report from America continent, Western Europe, Australia and East of Asia. Second, the sample size in some subgroup analysis was small (Yalin et al., 2007[35]; Tsai et al., 2011[33]; Grubisa et al., 2013[11]; Zaki et al., 2015[36]; Stoian et al., 2015[29]; Mergani et al., 2016[18]; Rasheed et al., 2016[23]; Ahmed and Al-Bachary, 2017[2]), which may increase the risk of false negatives or false positives. Third, there was no data regarding the source of control groups in studies used for meta-analysis (Moasser et al., 2012[19]; Grubisa et al., 2013[11]; Abbasi et al., 2014[1]; Zaki et al., 2015[36]; Stoian et al., 2015[29]; Mergani et al., 2016[18]; Rasheed et al., 2016[23]; Ahmed and Al-Bachary, 2017[2]). Therefore we failed to found source of heterogeneity. Fourth, there was still heterogeneity in the subgroup analysis. The more confounding factors should be considering. Finally, numerous environmental, genetic factors and the interactions among these factors contribute to the progression of T2DM. Our meta-analysis results did not adjust any confounding covariant, for example gender, drinking status, smoking habit. 

A more comprehensive analysis should be conducted when more original information is available and interactions among the risk factors are considered. Further well-designed large studies are required to investigate gene-environment interactions.

## Acknowledgement

This study was supported by Shiraz University.

## Conflict of interest

No competing interests are declared by any of the authors.

## Figures and Tables

**Table 1:**
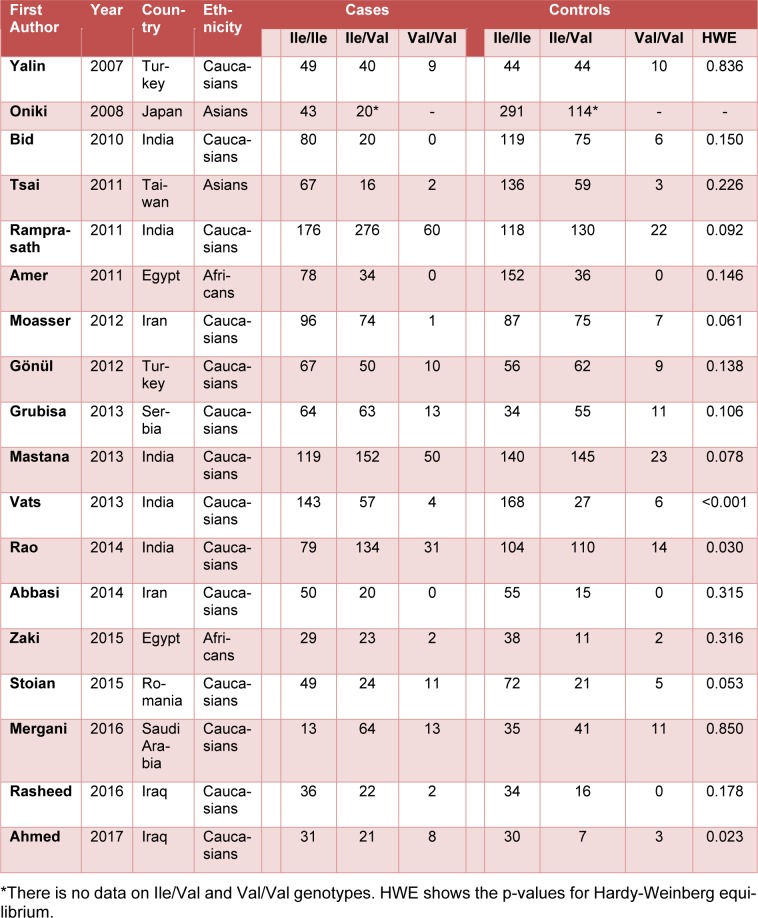
Characteristics of the studies included in the meta-analysis

**Table 2:**
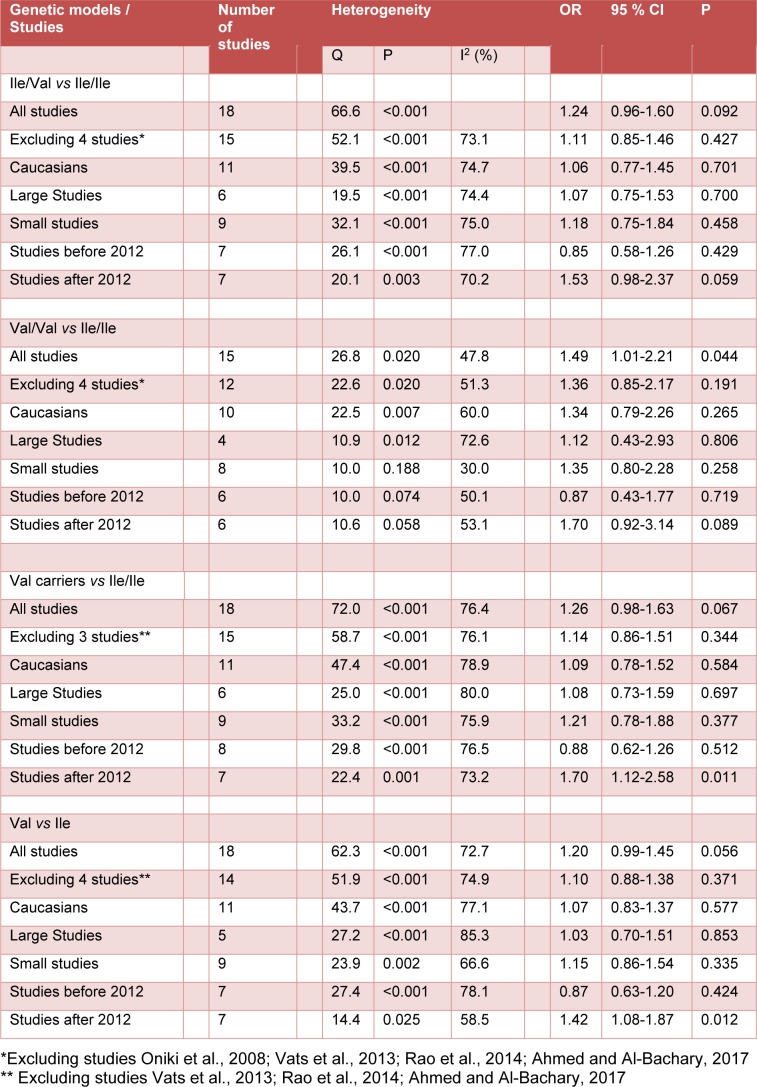
Summary of meta-analysis of studies evaluating *GSTP1* Ile105Val polymorphism and type 2 diabetes mellitus

## References

[1] Abbasi N, Salehi Z, Alizadeh Y (2014). Genetic variation of GSTP1 in diabetic retinopathy. Arak Med Uni J.

[2] Ahmed FAW, Al-Bachary HS (2017). Genetic polymorphism of glutathione S-transferase gene (GSTP1) in type 2 diabetes mellitus patients in Basra province/Iraq. Int J Sci.

[3] Almgren P, Lehtovirta M, Isomaa B, Sarelin L, Taskinen MR, Lyssenko V (2011). Heritability and familiality of type 2 diabetes and related quantitative traits in the Botnia Study. Diabetologia.

[4] Amer MA, Ghattas MH, Abo-Elmatty DM, Abou-El-Ela SH (2012). Evaluation of glutathione S-transferase P1 genetic variants affecting type-2 diabetes susceptibility and glycemic control. Arch Med Sci.

[5] Asmat U, Abad K, Ismail K (2016). Diabetes mellitus and oxidative stress-A concise review. Saudi Pharm J.

[6] Bid HK, Konwar R, Saxena M, Chaudhari P, Agrawal CG, Banerjee M (2010). Association of glutathione S-transferase (GSTM1, T1 and P1) gene polymorphisms with type 2 diabetes mellitus in north Indian population. J Postgrad Med.

[7] DerSimonian R, Laird N (1986). Meta-analysis in clinical trials. Contr Clin Trials.

[8] Egger M, Davey Smith G, Schneider M, Minder C (1997). Bias in meta-analysis detected by a simple, graphical test. Br Med J.

[9] Ginter E, Simko V (2012). Type 2 diabetes mellitus, pandemic in 21st century. Adv Exp Med Biol.

[10] Gönül N, Kadioglu E, Kocabaş NA, Ozkaya M, Karakaya AE, Karahalil B (2012). The role of GSTM1, GSTT1, GSTP1, and OGG1 polymorphisms in type 2 diabetes mellitus risk: a case-control study in a Turkish population. Gene.

[11] Grubisa I, Otasevic P, Despotovic N, Dedic V, Milašin J, Vucinic N (2013). Genetic polymorphism of glutathione S-treansferase P1 (GSTP1) Ile105Val and susceptibility to atherogenesis in patients with type 2 diabetes mellitus. Genetika.

[12] Hu X, Xia H, Srivastava SK, Herzog C, Awasthi YC, Ji X (1997). Activity of four allelic forms of glutathione S-transferase hGSTP1-1 for diol epoxides of polycyclic aromatic hydrocarbons. Biochem Biophys Res Commun.

[13] Jamhiri I, Saadat I, Omidvari S (2017). Genetic polymorphisms of superoxide dismutase-1 A251G and catalase C-262T with the risk of colorectal cancer. Mol Biol Res Commun.

[14] Johansson AS, Stenberg G, Widersten M, Mannervik B (1998). Structure-activity relationships and thermal stability of human glutathione transferase P1-1 governed by the H-site residue 105. J Mol Biol.

[15] Kang SW (2015). Superoxide dismutase 2 gene and cancer risk: evidence from an updated meta-analysis. Int J Clin Exp Med.

[16] Mantel N, Haenszel W (1959). Statistical aspects of the analysis of data from retrospective studies of disease. J Natl Cancer Inst.

[17] Mastana SS, Kaur A, Hale R, Lindley MR (2013). Influence of glutathione S-transferase polymorphisms (GSTT1, GSTM1, GSTP1) on type-2 diabetes mellitus (T2D) risk in an endogamous population from north India. Mol Biol Rep.

[18] Mergani A, Mansour AA, Askar T, Zahran RN, Mustafa AM, Mohammed MA (2016). Glutathione S-transferase Pi-Ile 105 Val polymorphism and susceptibility to T2DM in population from Turabah region of Saudi Arabia. Biochem Genet.

[19] Moasser E, Kazemi-Nezhad SR, Saadat M, Azarpira N (2012). Study of the association between glutathione S-transferase (GSTM1, GSTT1, GSTP1) polymorphisms with type II diabetes mellitus in southern of Iran. Mol Biol Rep.

[20] Oniki K, Umemoto Y, Nagata R, Hori M, Mihara S, Marubayashi T (2008). Glutathione S-transferase A1 polymorphism as a risk factor for smoking-related type 2 diabetes among Japanese. Toxicol Lett.

[21] Ramprasath T, Senthil Murugan P, Prabakaran AD, Gomathi P, Rathinavel A, Selvam GS (2011). Potential risk modifications of GSTT1, GSTM1 and GSTP1 (glutathione-S-transferases) variants and their association to CAD in patients with type-2 diabetes. Biochem Biophys Res Commun.

[22] Rao DK, Shaik NA, Imran A, Murthy DK, Ganti E, Chinta C (2014). Variations in the GST activity are associated with single and combinations of GST genotypes in both male and female diabetic patients. Mol Biol Rep.

[23] Rasheed RH, Al-Essa NE, Shihab BA (2016). Association of glutathione S-transferase (GSTP1) genetic polymorphism in Iraqi patients with diabetes mellitus type 2. Baghdad Sci J.

[24] Ryberg D, Skaug V, Hewer A, Phillips DH, Harries LW, Wolf CR (1997). Genotypes of glutathione transferase M1 and P1 and their significance for lung DNA adduct levels and cancer risk. Carcinogenesis.

[25] Saadat M (2006). Genetic polymorphisms of glutathione S-transferase T1 (GSTT1) and susceptibility to gastric cancer: a meta-analysis. Cancer Sci.

[26] Saadat M (2013). Null genotypes of glutathione S-transferase M1 (GSTM1) and T1 (GSTT1) polymorphisms increased susceptibility to type 2 diabetes mellitus, a meta-analysis. Gene.

[27] Saadat M, Saadat S (2015). Genetic polymorphism of CAT C-262 T and susceptibility to breast cancer, a case-control study and meta-analysis of the literatures. Pathol Oncol Res.

[28] Stančáková A, Laakso M (2016). Genetics of type 2 diabetes. Endocr Dev.

[29] Stoian A, Bănescu C, Bălaşa RI, Moţăţăianu A, Stoian M, Moldovan VG (2015). Influence of GSTM1, GSTT1, and GSTP1 polymorphisms on type 2 diabetes mellitus and diabetic sensorimotor peripheral neuropathy risk. Dis Markers.

[30] Sun X, Yu W, Hu C (2014). Genetics of type 2 diabetes: insights into the pathogenesis and its clinical application. Biomed Res Int.

[31] Sundberg K, Johansson AS, Stenberg G, Widersten M, Seidel A, Mannervik B (1998). Differences in the catalytic efficiencies of allelic variants of glutathione transferase P1-1 towards carcinogenic diol epoxides of polycyclic aromatic hydrocarbons. Carcinogenesis.

[32] Townsend DM, Tew KD (2003). The role of glutathione S-transferase in anti-cancer drug resistance. Oncogene.

[33] Tsai JP, Yang SF, Wu SW, Hung TW, Tsai HC, Lian JD (2011). Glutathione S-transferase gene polymorphisms are not major risks for susceptibility to post transplantation diabetes mellitus in Taiwan renal transplant recipients. J Clin Lab Anal.

[34] Vats P, Chandra H, Banerjee M (2013). Glutathione S-transferase and catalase gene polymorphisms with type 2 diabetes mellitus. Dis Mol Med.

[35] Yalin S, Hatungil R, Tamer L, Ates NA, Dogruer N, Yildirim H (2007). Glutathione S-transferase gene polymorphisms in Turkish patients with diabetes mellitus. Cell Biochem Funct.

[36] Zaki MA, Moghazy TF, El-Deeb MMK, Mohamed AH, Mohamed NAA (2015). Glutathione S-transferase M1, T1 and P1 gene polymorphisms and the risk of developing type 2 diabetes mellitus in Egyptian diabetic patients with and without diabetic vascular complications. Alexandria J Med.

[37] Zendehboodi Z (2017). Association of glutathione S-transferase M1 and T1 polymorphisms and temperament. Mol Biol Res Commun.

[38] Zimniak P, Nanduri B, Pikula S, Bandorowicz-Pikuła J, Singhal SS, Srivastava SK (1994). Naturally occurring human glutathione S-transferase GSTP1-1 isoforms with isoleucine and valine in position 104 differ in enzymic properties. Eur J Biochem.

